# Feasibility and Impact of the Combined Application of Coronary CT Angiography With the HEART Pathway in Patients With Suspected Acute Coronary Syndrome

**DOI:** 10.1097/HPC.0000000000000258

**Published:** 2021-03-01

**Authors:** Andrew J. Matuskowitz, Jihad S. Obeid, Lindsey Jennings, Richard R. Bayer, Viswanathan Ramakrishnan, U. Joseph Schoepf, Edward C. Jauch

**Affiliations:** From the * Department of Emergency Medicine, Medical University of South Carolina, Charleston, SC; † Department of Public Health Sciences, Medical University of South Carolina, Charleston, SC; ‡ Division of Cardiology, Department of Medicine, Medical University of South Carolina, Charleston, SC; § Division of Cardiovascular Imaging, Department of Radiology and Radiological Science, Medical University of South Carolina, Charleston, SC; ¶ Mission Research Institute, Mission Health, Asheville, NC.

**Keywords:** accelerated diagnostic protocol, chest pain, HEART Pathway, coronary CT angiography, clinical decision support

## Abstract

**Background:**

This study examines the feasibility and utility of integrating coronary computed tomography angiography and the HEART Pathway into a novel accelerated diagnostic protocol—called HEART-CT—and assesses its impact as an optional interactive decision support tool (smart form) in the electronic health record.

**Methods:**

This was a retrospective observational study performed in 2 adult emergency departments (ED) among patients evaluated for suspected acute coronary syndrome. Primary outcomes included the rate of discharge from the ED following HEART-CT Smart Form use, 30-day major adverse cardiac events (MACE), and ED length of stay (LOS). Hypothesis-generating outcomes included the rate of Smart Form use by ED providers and whether adhering to the HEART-CT recommendations was associated with improved outcomes.

**Results:**

The study included 672 subjects, 78.1% of whom were discharged from the ED. HEART-CT identified 76.7% of patients with increased risk HEAR scores as safe for discharge. No patients identified as low risk by HEART-CT had MACE within 30 days. Total mean ED LOS was 4.6 hours. ED providers used the HEART-CT smart form in 19.7% of eligible patients. ED providers who followed the HEART-CT recommendations had 3.41 times higher odds of ED discharging patients with increased risk HEAR scores than nonadherent providers (95% CI, 2.20-5.27).

**Conclusions:**

HEART-CT reclassified a large proportion of patients as safe for discharge, maintained a high sensitivity for detecting 30-day MACE, and had an acceptable ED LOS. Future studies should test the extent to which more automated clinical decision support improves provider adoption and clinical outcomes of HEART-CT.

## Abstract 2

Supplemental Digital Content is available in the text.

Annually in the United States, approximately 8 million patients present to the emergency department (ED) with the complaint of chest pain.^1^ Typically, more than half of these patients are admitted to the hospital or short-stay units for continued assessment of suspected acute coronary syndrome (ACS) but fewer than 10% are formally diagnosed with this life-threatening disease at discharge.^2^ Such over-triage strains hospital resources and exposes many patients to unnecessary cardiac testing and iatrogenic risks. Noninvasive coronary computed tomography angiography (CCTA) has demonstrated high negative predictive value in ruling out clinically significant obstructive coronary disease and maintains a higher sensitivity and specificity compared with myocardial perfusion stress testing.^3–5^ However, CCTA use without pretest risk stratification may increase cardiac testing, unnecessary radiation exposure, and healthcare costs with unclear benefits.^6,7^

The HEART Pathway is a commonly used accelerated diagnostic protocol (ADP) that identifies low-risk patients who can be safely discharged from the ED without further cardiac testing. It combines the HEAR score (*H*istorical features, *El*ectrocardiogram [ECG], *A*ge, and *R*isk factors) decision aid with serial 3-hour troponin concentrations to stratify patients into low- and high-risk groups.^8^ Patients identified by the HEART Pathway as low risk have less than 1% risk of major adverse cardiac events (MACE) at 30 days.^8,9^ Despite improved outcomes over usual care—which involves ECG interpretation, serial 3- to 6-hour troponin testing, and clinical gestalt—use of the HEART Pathway results in admission to inpatient or short-stay units in approximately 50% of patients, a disproportionately high percentage of whom are later found to have no evidence of clinically significant coronary artery disease.^8–10^ Previous studies have called for assessing the integration of CCTA with ED-based ADPs to reduce unnecessary cardiac testing in low-risk patients while more accurately stratifying increased risk patients that would benefit from hospitalization, yet no such investigations have been conducted.^11,12^

In an attempt to more accurately identify patients requiring hospitalization, we developed a novel clinical pathway called HEART-CT, which integrates CCTA with the HEART Pathway and embedded it into the electronic health record (EHR) as an interactive Smart Form for use by ED providers (Fig. 1 and Supplemental Figure 1 http://links.lww.com/HPC/A229). Smart Forms are EHR-based clinical decision support tools that enable provider documentation of clinical visits, promote structured data capture, and create actionable decision support in a single environment.^13^ The main objective of this study was to test the feasibility of implementing HEART-CT in the ED, specifically that its use would result in a high rate of discharges from the ED within an acceptable ED LOS and ensure 30-day MACE <1%. Additional hypothesis-generating objectives were to assess the extent to which a nonautomatically triggered clinical decision support tool integrated into the EHR would be used by ED providers and whether adhering to its recommendations in patients with increased risk (moderate or high) HEAR scores was associated with improved outcomes.

**FIGURE 1.**
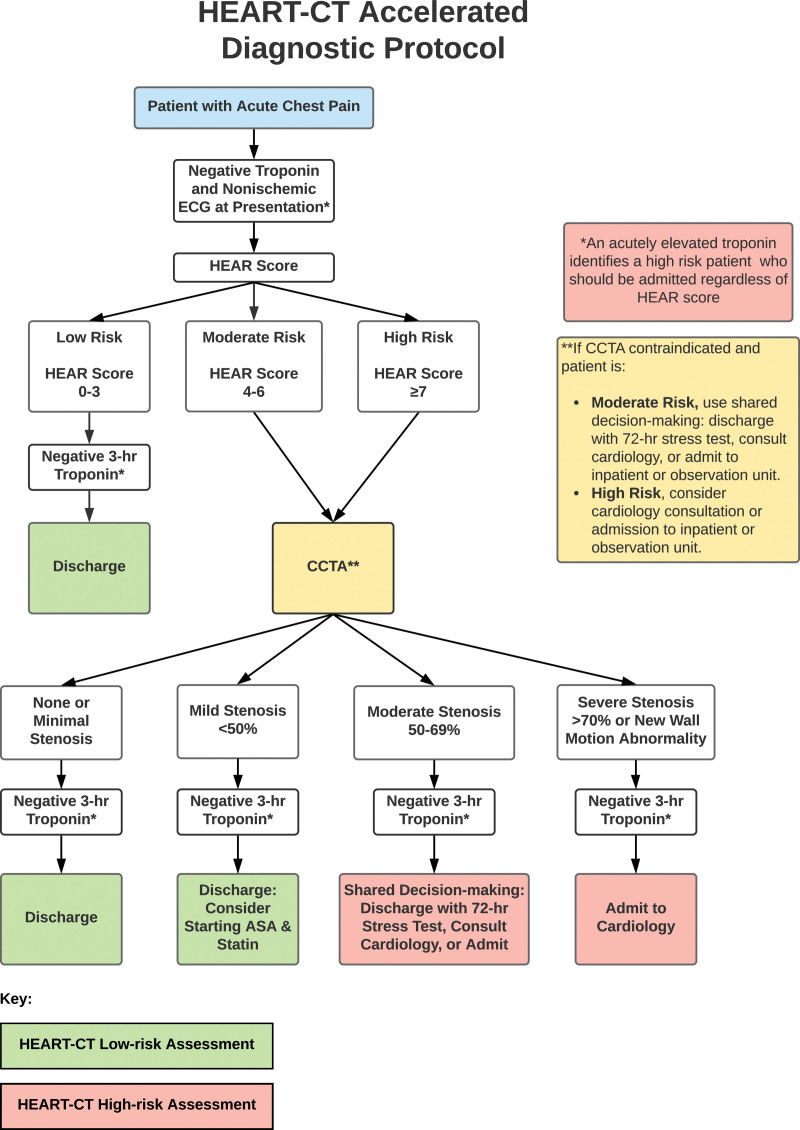
HEART-CT accelerated diagnostic protocol. CCTA indicates noninvasive coronary computed tomography angiography; ECG, electrocardiogram.

## METHODS

### Study Design and Setting

This was a retrospective observational study performed in 2 EDs associated with a tertiary care academic healthcare center from September 1, 2017, to December 27, 2018. The study was approved by the Institutional Review Board. The study institution served urban, suburban, and rural populations with an annual ED volume of approximately 60,000 patients per year. The EDs were staffed by board certified or board eligible Emergency Medicine physicians 24/7 who directly provided and supervised care provided by residents, physician assistants, physician assistant fellows, and nurse practitioners. Providers used institutionally accepted Abbott i-STAT (point-of-care) troponin I (99th percentile upper reference limit 0.08 ng/mL) or ARCHITECT troponin I (99th percentile upper reference limit 0.028 ng/mL) and were encouraged to utilize the same 3-hour troponin assay as the baseline assay. The cardiac testing modalities available to ED providers included a second or third-generation dual-source computed tomography scanner (Siemens Healthineers, Forchheim, Germany), offered 24/7, and nuclear myocardial perfusion stress testing during regular business hours. CCTA imaging was obtained either by dedicated CCTA alone or as part of a triple-rule-out CCTA protocol that additionally assessed for pulmonary emboli and thoracic aortic disease.^14^ Patients with acute chest pain were either admitted to the hospital or discharged following serial troponin measurements. There was no dedicated observation or short-stay unit at the medical center available for patients with increased risk HEAR scores.

### Selection of Participants

Inclusion criteria were as follows: adults at least 21 years of age presenting to the ED with a chief complaint of chest pain suggestive of ACS, an initial ED troponin result, and a completed HEART-CT Smart Form in the EHR. Exclusion criteria included patients with ST segment elevation myocardial infarction (STEMI) on initial ECG or any troponin level acutely above the 99th percentile upper reference limit during the index ED visit suggestive of acute myocardial injury. To reduce lost to follow-up bias, patient data were required from at least 2 separate medical center encounters within 2 years of data collection and the patient’s home addresses was within 20 miles of the study institution. A 20-mile radius was chosen to ensure a diverse inclusion of urban, suburban, and rural communities. Patients that were hospitalized within 30 days following the index visit were assessed for MACE. Repeat encounters that met inclusion criteria after 30 days were assessed for MACE but not added to the total number of patient encounters.

### Study Protocol and Measures

The HEART-CT clinical pathway was developed by emergency physicians in collaboration with cardiology and radiology physicians at the study institution in 2016. The HEART-CT Smart Form was embedded in the EHR in August 2017. HEART-CT Smart Form education was provided during Emergency Medicine resident didactic sessions and monthly faculty meetings over a 2-month period. Additionally, the HEART-CT ADP (Fig. 1) was uploaded to an easily accessible EHR-embedded webpage for supplemental reference.

Among patients for whom ACS was considered in the differential diagnosis, providers could choose to select the HEART-CT Smart Form located within a decision support tab separate from the ED provider documentation (Supplemental Figure 1, http://links.lww.com/HPC/A229). Utilization of the HEART-CT Smart Form was fully optional and there were no automated prompts to trigger its use. In the Smart Form, providers selected pertinent positive elements of the HEAR score, which generated a final component score. HEAR score elements were described previously and are included in Supplemental Figure 2, http://links.lww.com/HPC/A230.^8^ In patients with low-risk HEAR scores, the Smart Form generated an automated response recommending discharge from the ED following a nonischemic ECG and normal serial troponin results. In patients with moderate- or high-risk HEAR scores, the Smart Form generated follow-up questions assessing whether CCTA was contraindicated. CCTA was considered contraindicated in the following scenarios: creatinine ≥2.0 mg/dL, weight >300 pounds, or active atrial fibrillation. If not contraindicated, the Smart Form generated an automated response recommending CCTA immediately following a nonischemic ECG and initial normal troponin result. Additionally, CCTA was recommended as part of a triple-rule-out assessment if there was increased clinical suspicion for pulmonary embolism or aortic dissection.^15^ If CCTA was contraindicated, the Smart Form recommended cardiology consultation, hospital admission, or discharge with a 72-hour stress test for patients with moderate-risk HEAR scores and cardiology consultation or hospitalization for patients identified with high-risk HEAR scores.

A final HEART-CT risk assessment was determined by combining the HEAR score generated from the HEART-CT Smart Form and final imaging results among those who received CCTA. Specifically, HEART-CT dichotomized patients into low-risk and high-risk categories based on the following (see also Fig. 1):

- Low-risk HEART-CT: patients with (1) low-risk HEAR scores or (2) increased risk HEAR scores (moderate or high) that were followed by one of the following CCTA results: none, minimal, or mild coronary stenosis.^16^
- High-risk HEART-CT: patients with (1) increased risk HEAR scores and (2) moderate or severe coronary stenosis by CCTA.^10,16^

Additionally, the Smart Form recommended hospital admission for any patient with a troponin level acutely above the 99th percentile upper reference limit or acute ischemic ECG findings. ED providers were instructed during didactics and faculty meetings to avoid HEART-CT use in patients with prior acute myocardial infarction (AMI), coronary stents, or coronary bypass grafts as these patients represent a higher risk population outside the scope of this ADP.

Data abstractors (AM and LJ) used a standardized REDCap (Research Electronic Data Capture) data extraction form for data capture and management. Data abstraction were duplicated on 30 patients to calculate interobserver agreement. The following information were collected by the data query: patient demographics, disposition during the index visit, any hospitalization within 30 days of the index visit, total component HEAR score, CCTA results, ED LOS, and troponin results. To classify CCTA results into discreet categories of stenosis and identify any possible MACE outside of the index ED visit, manual chart review was conducted on patients who underwent CCTA or hospitalized during the index visit or within 30 days. Manual chart review was not performed on patients discharged from the ED during the index visit without a 30-day hospitalization because we reasoned that MACE would have been very unlikely to occur in this low-risk group. Additionally, the Social Security Death Index was queried through the South Carolina Department of Health and Environmental control to identify mortality that was not captured by the EHR at our institution. Data abstractors identified instances of MACE defined as AMI, coronary revascularization during the index visit or within 30 days, and death by cardiovascular or noncardiovascular cause. Researchers classified the degree of coronary stenosis on CCTA as none or minimal, mild (<50%), moderate (50–69%), or severe (>70%) based on the most stenosed vessel reported.^17^ AMI was based on the 2018 American College of Cardiology/European Society of Cardiology/American College of Cardiology/American Heart Association/World Heart Federation consensus definition.^18^ Adherence to HEART-CT recommendations was defined as (1) patients with low-risk HEAR scores that did not receive CCTA and (2) patients with moderate or high-risk HEAR scores that received CCTA. Nonadherence was defined as (1) patients with low-risk HEAR scores that received CCTA and (2) patients with moderate or high-risk HEAR scores that did not receive CCTA.

### Primary and Secondary Outcomes

In this feasibility study, the primary outcome was rate of discharge from the ED following HEART-CT use. The secondary outcomes were MACE within 30 days and ED LOS. Additional hypothesis-generating outcomes were the rate of HEART-CT Smart Form use relative to the total number of patients presenting to the ED with chest pain and the association between following the HEART-CT recommendations in patients with increased risk HEAR scores and discharge rate, MACE, and ED LOS.

### Statistical Analyses

All variables except ED LOS were categorical. These included HEAR score risk classification, degree of coronary stenosis by CCTA, troponin concentration (classified as above or below the 99th percentile upper reference limit), HEART-CT risk classification, index visit disposition, HEART-CT adherence, and MACE. MACE was subdivided into index visit MACE and 30-day MACE, each of which included AMI, revascularization without AMI, cardiovascular death, and noncardiovascular death. All analyses were carried out using SPSS Statistics V25 (IBM, Armonk, NY). Comparisons of categorical variables were performed using the *X*^2^ test. LOS comparisons were performed using the *t* test assuming independent samples. Significant differences in baseline characteristics were further assessed by MACE between groups using *X*^2^ tests. The Fisher exact test was used for any observations less than 5. Odds ratios for HEART-CT provider adherence versus nonadherence in patients with increased risk HEAR scores were carried out by logistic regression. Since age is a predictor variable in the HEAR score, it was not independently analyzed. The Strengthening the Reporting of Observational Studies in Epidemiology (STROBE) guidelines were adhered to in reporting this study and its results.^19^

## RESULTS

### Characteristics of Study Subjects

**FIGURE 2.**
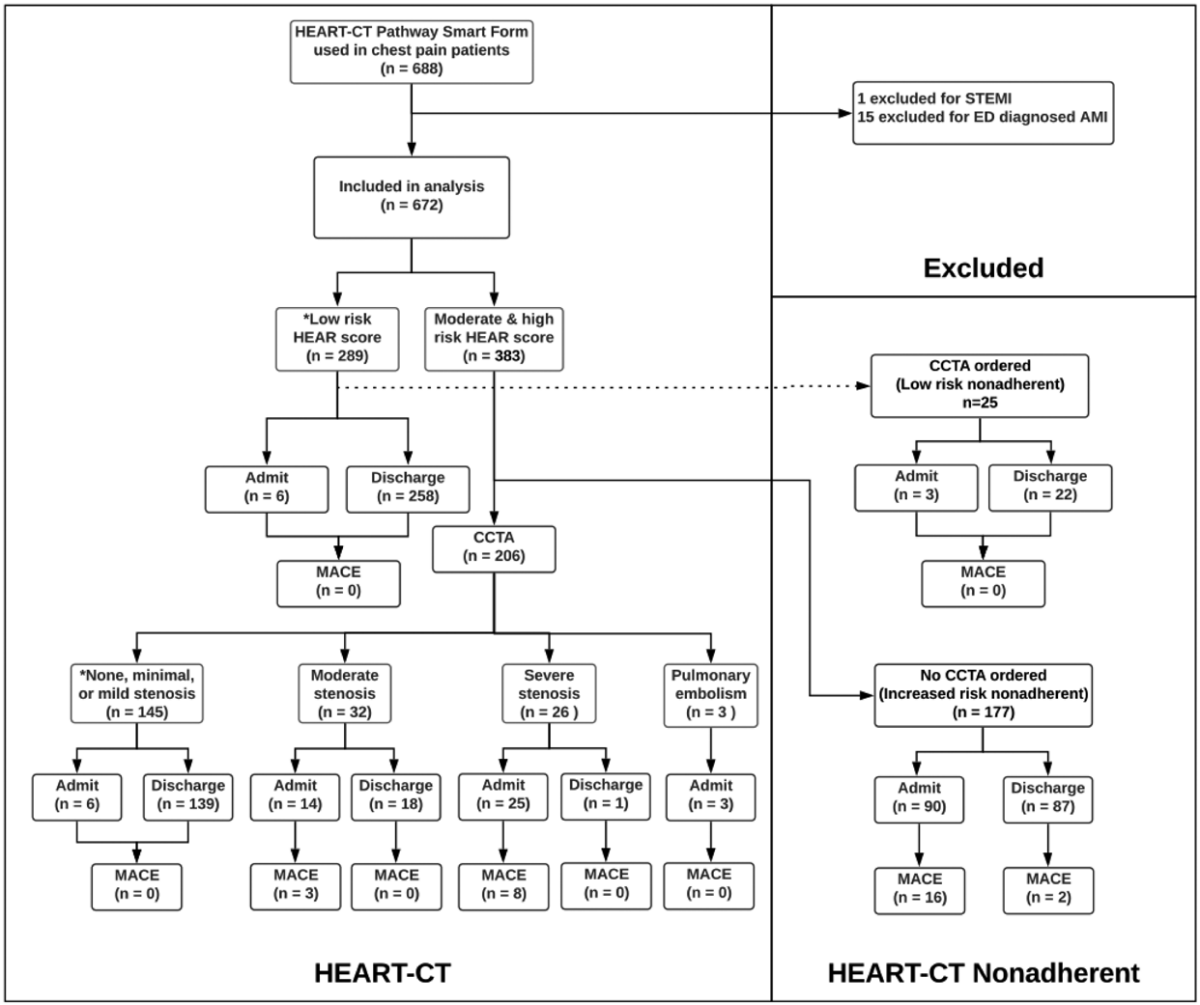
Flow diagram of the study cohort used for analysis. *HEART-CT low-risk assessment. AMI indicates acute myocardial infarction; CCTA, noninvasive coronary computed tomography angiography; ED, emergency departments; MACE, major adverse cardiac event; STEMI, ST segment elevation myocardial infarction.

From September 1, 2017, to December 27, 2018, the HEART-CT Smart Form was utilized 733 times in 688 patients. One patient was excluded for a STEMI and 15 for troponin elevations in the ED, each of which was diagnosed as an AMI. The remaining 672 patients were included in the analysis (Fig. 2). Manual chart review was conducted on 337 patient encounters (Supplemental Table 1, http://links.lww.com/HPC/A231). There were no missing data elements. Patient characteristics are included in Table 1. There were more female than male and more black than white patients. Since males were more likely than females to have a high-risk HEART-CT assessment (15.1% vs. 9.0%, *P* = 0.036), a bivariate regression analysis was conducted and revealed no difference in MACE between males and females with high HEART-CT risk (18.2% vs. 20.0%, *P* = 0.845) (Supplemental Tables 2A and 2B, http://links.lww.com/HPC/A232). There were no significant differences between black, white, and other races, or between Hispanic and non-Hispanic patients. Interobserver agreement among blinded abstractors for identifying MACE was high (kappa = 0.83 and 1.00 for index visit MACE and 30-day MACE, respectively).

**TABLE 1.** Patient Characteristics

Patient Variables	Total (N = 672)
Age – mean ± SD	54.3 ± 15.2
Male gender	298 (44.3)
Race and ethnicity	
Black or African American	351 (52.2)
White or Caucasian	297 (44.2)
Asian	2 (0.3)
Hispanic or Latino	15 (2.2)
Other	7 (1.0)
HEAR score	672 (100.0)
Low risk	289 (43.0)
Moderate risk	348 (51.8)
High risk	35 (5.2)
CCTA or TRO	231 (34.4)
None or minimal	146 (21.7)
Mild	19 (2.8)
Moderate	34 (5.1)
Severe	28 (4.2)
Pulmonary embolism	4 (0.6)
Aortic disease	0 (0)
HEART-CT risk	
Low risk	437 (65.0)
High risk	58 (8.6)
Disposition (index visit)	
Discharged	525 (78.1)
Admitted	147 (21.9)

Data are presented as No. (%).

CCTA indicates noninvasive coronary computed tomography angiography; TRO, triple-rule-out.

### Main Results

Table 2 summarizes the primary outcome. Of 672 patients included in the study, 78.1% were discharged from the ED. Adhering to the HEART-CT recommendations reclassified 76.7% (158/206) of patients with increased risk HEAR scores (moderate or high) as safe for discharge following nonobstructive CCTA results. Tables 3 and 4 summarize the secondary safety outcomes. MACE occurred in 4.3% (29/672) of the cohort and in 7.6% (29/383) of patients with increased risk HEAR scores. No patients identified as low risk by HEART-CT during the index visit and discharged from the ED had MACE within 30 days (0/437). In contrast, 19% (11/58) of patients identified as high risk by HEART-CT had MACE within 30 days. The sensitivity and specificity of HEART-CT was 100% and 90.3%, respectively. The positive predictive value and negative predictive of HEART-CT was 19.0% and 100%, respectively. The mean ED LOS was 4.62 ± 3.67 hours. Among providers who adhered to the HEART-CT recommendations, CCTA use in increased risk patients increased ED LOS by 2.26 hours compared with low-risk patients who did not receive CCTA (95% CI, 1.70-2.82).

**TABLE 2.** Disposition by HEAR Score and HEART-CT Adherence

HEAR score	Admitted	Discharged	Total
Low-risk HEAR score			
Overall	9 (3.1)	280 (96.9)	289
HEART-CT adherent	6 (2.3)	258 (97.7)	264
Nonadherent	3 (12.0)	22 (88.0)	25
Moderate-risk HEAR score			
Overall	109 (31.3)	239 (68.7)	348
HEART-CT adherent	46 (22.8)	156 (77.2)	202
Nonadherent	63 (43.2)	83 (56.8)	146
High-risk HEAR score			
Overall	29 (82.9)	6 (17.1)	35
HEART-CT adherent	2 (50.0)	2 (50.0)	4
Nonadherent	27 (87.1)	4 (12.9)	31
Total			
Overall	147 (21.9)	525 (78.1)	672
HEART-CT adherent	54 (11.5)	416 (88.5)	470
Nonadherent	93 (46.0)	109 (54.0)	202

Data are presented as No. (%).

**TABLE 3.** MACE Outcomes by HEAR Score and CCTA

Stratification Technique	MACE	No MACE	Total
**HEAR Score**	**N = 29**	**N = 643**	**N = 672**
Low-risk HEAR score	0 (0)	289 (100)	289
Moderate-risk HEAR score	21 (6.0)	327 (94.0)	348
High-risk HEAR score	8 (22.9)	27 (77.1)	35
**CCTA**	**N = 11**	**N = 220**	**N = 231**
None, minimal, or mild stenosis	0 (0)	165 (100)	165
Moderate stenosis	3 (8.8)	31 (91.2)	34
Severe stenosis	8 (28.6)	20 (71.4)	28
Pulmonary embolism	0 (0)	4 (100)	4

Data are presented as No. (%).

CCTA indicates noninvasive coronary computed tomography angiography.

**TABLE 4.** MACE Outcomes by HEART-CT Risk Assessment

Risk	MACE (N = 11)	No MACE (N = 484)	Total (N = 495)	*P*
Increased risk	11 (19)	47 (81)	58	<0.001
Low risk	0 (0)	437 (100)	437	
Sensitivity	100%			
Specificity	90.3%			
Positive predictive value	19.0%			
Negative predictive value	100%			

Data are presented as No. (%) unless otherwise noted.

MACE indicates major adverse cardiac events.

Among the hypothesis-generating outcomes, the HEART-CT Smart Form was utilized in 19.7% (688/3488) of patients presenting with chest pain who had a documented initial ED troponin result. Table 5 summarizes outcome comparisons of subjects with increased risk HEAR scores based on adherence to HEART-CT recommendations. Among patients with increased risk HEAR scores, ED providers that followed the HEART-CT recommendations had 3.41 times higher odds of discharging patients than nonadherent providers (95% CI, 2.20-5.27). There were no differences detected in MACE rates (OR, 2.01; 95% CI, 0.92-4.37) or ED LOS (−0.43 h; 95% CI, −0.92 to 0.83) when providers were adherent versus nonadherent, respectively.

**TABLE 5.** Outcome Comparisons of Patients With Increased Risk HEAR Scores (Moderate and High) Based on Adherence to HEART-CT

Outcome	Adherent (N = 206)	Nonadherent (N = 177)	OR/Difference	95% CI
MACE	11 (5.3)	18 (10.2)	OR, 2.01	0.92, 4.37
ED discharged	158 (76.7)	87 (49.2)	OR, 3.41	2.20, 5.27
Mean length of stay (in hours)	5.50	5.55	Difference, -0.43	-0.92, 0.83

Data are presented as No. (%).

CI indicates confidence interval; ED, emergency department; MACE, major adverse cardiac events; OR, odds ratio.

## DISCUSSION

This is among the first studies to assess the feasibility and impact of the combined application of CCTA and the HEART Pathway and the first to do so with an electronic decision support tool.^9,20,21^ There are 4 notable findings as a result of this work. First, 78.1% of the patient cohort was discharged from the ED, and HEART-CT reclassified 76.7% of patients with increased risk HEAR scores as low risk without increasing MACE or ED LOS. Second, no patients who were identified as low risk by HEART-CT had a 30-day MACE. Third, mean ED LOS was acceptable, even in patients who underwent CCTA. Fourth, provider education and fully optional decision support tools did not sufficiently ensure adherence to the novel ADP, but when used, may be associated with increased ED discharges.

This study builds on recent findings that CCTA use in HEART score derived ADPs significantly reduces unnecessary hospitalizations. For instance, Arslan et al reclassified 74.1% of patients with intermediate HEART scores as low risk using CCTA.^20^ Shin et al similarly reclassified 68.7% as low risk, 28.7% more than the HEART Pathway alone.^21^ Our collective findings are important given that that only 36% of US hospitals staff dedicated observation units according to the latest National Hospital Ambulatory Medical Care Survey.^22^ The remaining majority of hospitals without observation units or an in-house cardiology team may particularly benefit from an algorithm that integrates a chest pain ADP with CCTA for increased risk patients.

Our study expands the generalizability of Shin et al and Arslan et al’s findings by exploring an American, majority black population. Also, those studies assessed HEART scores only in patients who underwent CCTA, excluding a significant proportion of low-risk patients.^20,21^ Our study included all low-risk patients, which more closely aligns with real-world scenarios in which ED providers will use the HEAR score to determine whether CCTA is appropriate.

The risks associated with CCTA must be weighed against each patient’s MACE risk. Zero out of 289 low-risk patients in our study had 30-day MACE, which supports avoiding additional cardiac testing in this subgroup. However, nearly 8% of patients with increased risk HEAR scores had MACE within 30 days, justifying CCTA use in this subgroup.

The HEART-CT Smart Form was more passive than active, located in a separate decision support tab within the EHR and not automatically triggered by discreet patient data. The relatively low proportion HEART-CT Smart Form use in our study is consistent with previous findings that without automated provider alerts in the EHR, education alone may be insufficient to promote widespread use of ADPs.^23^ Effective implementation requires a multimodal approach that maximizes usability. Important steps for successful deployment include appropriate timing of the guideline trigger, automated, or semiautomated algorithms that provide real-time recommendations to end-users, minimally disruptive provider workflows, and usefulness of the content to end-users.^13,23–25^ In contrast to our first attempt at EHR-integrated decision support, Smulowitz et al demonstrated 72% adherence to the HEART Pathway by flagging providers with best practice alerts for patients that met HEAR score criteria based on a chief complaint of chest pain, age ≥30, and a negative first troponin result.^9^ The authors also found that deployment of their EHR-embedded HEART Pathway reduced admissions by approximately 8%. Widespread adoption of HEART-CT would require development of more automated clinical decision support tools that can be readily shared across EHRs. Thus, future studies should test the extent to which automated EHR-embedded clinical decision support improves adherence to and clinical outcomes of HEART-CT.

The pragmatic nature of our study design included some limitations. First, the optional HEART-CT Smart Form was only completed on 19.7% of eligible patients presenting with chest pain. The remaining 80.3% of patients may theoretically have represented a different patient risk group. However, the incidence of 30-day MACE in our sample was similar to that of other HEART Pathway studies, suggesting that our sample represents an appropriate distribution of ACS risk.^10,26^ Second, we did not formally assess whether ED providers excluded patients from the HEART-CT Smart Form with prior AMI, coronary stents, or coronary bypass grafts, which would have resulted in higher risk patients inappropriately receiving HEAR scores or CCTA. However, if such patients were included, our 100% sensitivity suggests that none of these patients had an adverse outcome associated with inaccurate risk classification. Third, the higher rate of admissions observed in increased risk patients who did not undergo CCTA may represent a higher risk patient population than those identified as HEART-CT adherent. It is possible that many of these patients may have had contraindications to CCTA use, including chronic renal disease, tachycardia, or prior cardiac stents or coronary bypass grafting. Controlling for these potential selection biases will be important in prospective testing of HEART-CT. An additional limitation of our study is that retrospective chart review may have increased follow-up bias risk. To reduce the risk of missing MACE, however, we queried the social security death index and only included patients who resided within a 20-mile radius and had at least 2 visits (i.e., ED, inpatient, or outpatient clinic) at our medical center within 2 years of data collection.

## CONCLUSIONS

Our findings suggest that HEART-CT reclassifies a large proportion of patients with increased risk HEAR scores as safe for discharge from the ED while maintaining an acceptable ED LOS. Improved diagnostic precision using HEART-CT may reduce inpatient overcrowding in many cases and the need for admission to observation units in others. Prospective validation of HEART-CT in other ED settings is needed to determine whether a paradigm shift focusing on rapid imaging of appropriately selected patients is justified. Importantly, EHR-integrated clinical decision support without automated triggers may be insufficient to ensure widespread adoption of such an ADP. Thus, the development of more automated clinical decision support tools is needed to assess the impact of EHR-integrated clinical decision support on HEART-CT.

## ACKNOWLEDGMENTS

The authors thank all members of the study institution who made this project possible, especially the emergency medicine, cardiology, and radiology attendings, residents, physician assistants, physician assistant fellows, nurse practitioners, nursing staff, and ancillary staff who directly or indirectly provide care to patients. The authors also thank Dr. Simon Mahler, MD, MS, who provided guidance and clarification on the HEART Pathway accelerated diagnostic protocol. Finally, the authors thank the National Center for Advancing Translational Sciences of the National Institutes of Health and the National Institute of General Medical Sciences for research support.

## DISCLOSURES

*Dr. Matuskowitz receives institutional research support from HeartFlow Inc. Dr. Bayer receives institutional research support from Bayer, HeartFlow Inc. and Siemens Healthineers. Dr. Schoepf receives institutional research support from and/or is a consultant for Astellas, Bayer, Bracco, Elucid BioImaging, General Electric, HeartFlow Inc., Keya Medical, and Siemens Healthineers. The other authors have no conflicts of interest to disclose. This project was supported in part by the National Center for Advancing Translational Sciences of the National Institutes of Health under Grant Number UL1 TR001450 and the National Institute of General Medical Sciences under Grant Numbers U54-GM104941 and P20-GM109040. The content is solely the responsibility of the authors and does not necessarily represent the official views of the National Institutes of Health.*

## Supplementary Material


