# Supplementary material for: Feasibility and Impact of the Combined Application of Coronary CT Angiography With the HEART Pathway in Patients With Suspected Acute Coronary Syndrome
Source: Crit Pathw Cardiol. 2021 Mar 1;20(4):185–91. doi: 10.1097/HPC.0000000000000258 (PMC8408286; doi:10.1097/HPC.0000000000000258)
Supplement: Supplementary file 1 [file hpc-20-185-s001.pdf]

Supplemental Materials

Supplemental Figure 1. HEART-CT Smart Form.

History

2-->Mostly high-risk features | 1-->Mixture of high and low-risk features | 0-->Mostly low-risk features

High-risk Features	Low-risk Features
- Middle- or left-sided<br>- Heavy chest pain<br>- Diaphoresis<br>- Radiation<br>- N/V<br>- Exertional	- Well localized<br>- Sharp pain<br>- Non-exertional<br>- No diaphoresis<br>- No N/V

EKG

2-->Ischemic changes | 1-->Nonspecific changes | 0-->Normal

Normal	Nonspecific changes	Ischemic changes
- No ST segment or T wave changes	- Repolarization abnormalities<br>- Non-specific T wave changes<br>- Non-specific ST-segment depression or elevation<br>- Bundle branch blocks<br>- LVH<br>- Digoxin effect	- New ST-segment depressions<br>- New T-wave inversions

Age

2--> >65yrs old | 1--> 45-65yrs old | 0--> <45yrs old

Risk Factors

2-->Three or more risk factors or history of CAD | 1-->One or two risk factors | 0-->No risk factors known

Risk factors include	Atherosclerotic disease includes
- Hypercholesterolmia<br>- Hypertension<br>- Diabetes Mellitus<br>- Cigarette smoking (in last 1 month)<br>- Positive family history<br>- Obesity	- Myocardial infarction<br>- Coronary revascularization<br>- Stroke<br>- Peripheral arterial disease

Troponin

2-->Elevated troponin | 0-->Normal troponin

HEART Score Total

4

HEART Score 1-3 = Low Risk Chest Pain. Patient has approximately 0.6% risk of having an adverse cardiac event within 30 days if serial troponins are negative. In general, patient can be discharged without further cardiac testing at this time unless otherwise noted.
HEART Score 4-6 = Moderate Risk Chest Pain. Patient has significant risk of adverse cardiac event within 30 days.
HEART Score ≥ 7 = High Risk. Patient should be admitted for cardiac workup.
Reference: Crit Pathw Cardiol. 2011 Sep;10(3):128-33

Prior CABG or coronary stent?

Yes | No

Does the patient have any of the following?

Yes | No

- HR persistently > 90 or active A-fib
- Weight > 300 lbs
- Pulmonary edema
- Difficulty holding breath > 15 sec
- CrCl < 30 (Exception: chronic dialysis patient *can* receive IV contrast)
- Contrast allergy

Choose one of the following:

Coronary CTA (Preferred modality) - Coronary CTA was ordered to evaluate for significant CAD. | Triple Rule Out
