# Supplementary material for: Feasibility and Impact of the Combined Application of Coronary CT Angiography With the HEART Pathway in Patients With Suspected Acute Coronary Syndrome
Source: Crit Pathw Cardiol. 2021 Mar 1;20(4):185–91. doi: 10.1097/HPC.0000000000000258 (PMC8408286; doi:10.1097/HPC.0000000000000258)
Supplement: Supplementary file 2 [file hpc-20-185-s002.pdf]

Supplemental Figure 2. HEAR score decision aid.

HEAR Score

Note:

- HEAR score should be included with 0- and 3-hour troponins. Patients with an acutely elevated troponin or new ischemic ECG findings should be hospitalized.
- Hospitalization or Cardiology consultation is recommended in patients with prior MI, coronary stent, or CABG.

History

High-risk Features	Low-risk Features
▪ Middle- or left-sided<br>▪ Heavy chest pain<br>▪ Diaphoresis<br>▪ Radiation<br>▪ N/V<br>▪ Exertional	▪ Well localized<br>▪ Sharp pain<br>▪ Non-exertional<br>▪ No diaphoresis<br>▪ No N/V

Highly suspicious	**2 points**	Mostly high-risk features
Moderately suspicious	**1 point**	Mixture of high- and low-risk features
Slightly suspicious	**0 points**	Mostly low-risk features

ECG

New ischemic changes	**Increased mortality risk. Admit patient.**	• Ischemic ST-segment depression<br>• New ischemic T-wave inversions
Non-specific changes	**1 point**	• Repolarization abnormalities<br>• Non-specific ST-segment depression or elevation<br>• Bundle branch blocks<br>• Pacemaker rhythms<br>• LVH<br>• Early repolarization<br>• Digoxin effect
Normal	**0 point**	• Completely normal

Age

Age	**2 points**
≥ 65	**1 point**
45-64	**0 points**

Risk Factors

- Obesity (BMI ≥ 30)
- Current or recent ≤ 90 days) smoker
- Currently treated diabetes mellitus
- Family history of CAD (1st degree relative < 55 y.o.)
- Diagnosed and/or treated hypertension
- Hypercholesterolemia
- Atherosclerotic disease (Known CAD, prior CVA/TIA, peripheral arterial disease)

≥ 3 risk factors or history of atherosclerotic disease	**2 points**
1-2 risk factors	**1 point**
No risk factors	**0 points**

BMI, body mass index; *CAD*, coronary artery disease; *CVA*, cerebrovascular accident; *LVH*, left ventricular hypertrophy; *N/V*, nausea and vomiting; *TIA*, transient ischemic attack
