# Supplementary material for: Feasibility and Impact of the Combined Application of Coronary CT Angiography With the HEART Pathway in Patients With Suspected Acute Coronary Syndrome
Source: Crit Pathw Cardiol. 2021 Mar 1;20(4):185–91. doi: 10.1097/HPC.0000000000000258 (PMC8408286; doi:10.1097/HPC.0000000000000258)
Supplement: Supplementary file 3 [file hpc-20-185-s003.pdf]

Supplemental Table 1. Criteria for manual chart review: any patient admitted during the index visit or within 30-days or had CCTA performed during the index visit.

**Criteria**			**Charts Reviewed**	**Comment**
**HEAR Score Risk**	**Index visit Disposition**	**CCTA Use**		
Low	Admitted	Yes	3	
Low	Admitted	No	6	
Low	Discharged	Yes	22	
Low	Discharged	No	N/A	No 30-day hospitalizations triggered by data query.
Increased	Admitted	Yes	48	
Increased	Admitted	No	90	
Increased	Discharged	Yes	158	
Increased	Discharged	No	2	Identified by 30-day rehospitalization
**Other**			8	Discharged with no CCTA but had 30-day hospitalization not identified by other characteristics above
**Total**			337	

Data are presented as No.

CCTA, noninvasive coronary CT angiography
