# Supplementary material for: Feasibility and Impact of the Combined Application of Coronary CT Angiography With the HEART Pathway in Patients With Suspected Acute Coronary Syndrome
Source: Crit Pathw Cardiol. 2021 Mar 1;20(4):185–91. doi: 10.1097/HPC.0000000000000258 (PMC8408286; doi:10.1097/HPC.0000000000000258)
Supplement: Supplementary file 4 [file hpc-20-185-s004.pdf]

Supplemental Table 2A. Patient characteristics stratified by Heart-CT risk assessment.

**Characteristics**	**Low-risk** ***Number of patients (%)***	**High-risk** ***Number of patients (%)***	**P-value**
Sex			
Female	252 (91.0)	25 (9.0)	0.036
Male	185 (84.9)	33 (15.1)	
Race			
Black	241 (90.9)	24 (9.1)	0.183
White	176 (84.2)	33 (15.8)	
Other	20 (95.2)	1 (4.8)	
Ethnicity			
Hispanic	11 (100)	0 (0)	0.117
Not Hispanic or Latino	425 (88.2)	57 (11.8)	
Unknown	1 (50.0)	1 (50.0)	

Data are presented as No. (%).

Supplemental Table 2B: MACE outcomes by gender stratified into HEART-CT risk groups.

**Characteristics**	**MACE** ***Number of patients (%)***	**No MACE** ***Number of patients (%)***	**P-value**
Females			
Low-risk	0 (0)	252 (100)	< 0.001
High-risk	5 (20.0)	20 (80.0)	
Males			
Low-risk	0 (0)	185 (100.0)	< 0.001
High-risk	6 (18.2)	27 (81.8)	

Data are presented as No. (%).
